# Material Science in Cervical Total Disc Replacement

**DOI:** 10.1155/2015/719123

**Published:** 2015-10-07

**Authors:** Martin H. Pham, Vivek A. Mehta, Alexander Tuchman, Patrick C. Hsieh

**Affiliations:** Department of Neurosurgery, Keck School of Medicine, University of Southern California, Los Angeles, CA 90033, USA

## Abstract

Current cervical total disc replacement (TDR) designs incorporate a variety of different biomaterials including polyethylene, stainless steel, titanium (Ti), and cobalt-chrome (CoCr). These materials are most important in their utilization as bearing surfaces which allow for articular motion at the disc space. Long-term biological effects of implanted materials include wear debris, host inflammatory immune reactions, and osteolysis resulting in implant failure. We review here the most common materials used in cervical TDR prosthetic devices, examine their bearing surfaces, describe the construction of the seven current cervical TDR devices that are approved for use in the United States, and discuss known adverse biological effects associated with long-term implantation of these materials. It is important to appreciate and understand the variety of biomaterials available in the design and construction of these prosthetics and the considerations which guide their implementation.

## 1. Introduction

Total disc replacement (TDR) was initially developed as an alternative to fusion with the aim of preserving segmental motion. Cervical TDR has been used following an anterior discectomy for the treatment of radiculopathy or myelopathy. Although the anterior cervical discectomy and fusion (ACDF) has been successful with regard to overall outcome, fusion does lead to increased biomechanical stress at adjacent segments that may then accelerate degeneration at these levels [1–5]. Arthroplasty preserves the motion at the operated level and should reduce the rate of adjacent level pathology as well as avoid any complications associated with pseudoarthrosis.

The past several years have seen the continued research and development of suitable materials for arthroplasty. Current cervical TDR designs constitute a wide range of biomaterials available for their construction. The most common design used includes metallic endplates which are fixed to the vertebral bodies above and below, with one or more articulations that involve metal-on-metal or metal-on-polymer bearing surfaces at the central core [6]. A broad range of materials are used in the cervical spine and include polyethylene, cobalt-chrome (CoCr) alloys, stainless steel, titanium (Ti) alloys, polyurethanes, and Ti alloy-ceramic composites. The choice of biomaterials utilized in these prosthetic implants centers around their sufficient durability, biocompatibility, and resistance to mechanical loading during physiologic use [7].

In this paper, we provide a review of the biomaterials used in cervical TDR devices, describe the type of bearing designs and their material considerations, review the construction of the seven current cervical TDR prosthetic implants that are approved for use in the United States by the Food and Drug Administration (FDA), and describe known adverse biological effects associated with the implantation of these materials.

## 2. Materials

The choice of materials used in a prosthesis takes into consideration those used in the articulation surfaces as well as outer surfaces of the prosthesis that interface with the endplates of the vertebral bodies themselves. The bearing surfaces must be made of materials to tolerate loading without fatigue or fracture, minimize friction, have superior wear characteristics, and minimize the generation of wear debris [8].

Articular surfaces may use components made from polymers such as ultrahigh molecular weight polyethylene (UHMWPE). Metals also play a key role in implant design and creation. Metallic components have been utilized which may wear more slowly than UHMWPE and include stainless steel, titanium (Ti), and cobalt-chrome (CoCr) alloy.

Although the initial stability of an artificial disc depends on soft tissue tensioning and implant design and geometry, long-term fixation depends on bony ingrowth into the surface of the prosthesis. Surface coatings have been used to improve this type of bony ingrowth and include titanium wire mesh, plasma-sprayed titanium, porous CoCr, and bioactive materials such as hydroxyapatite and calcium phosphate [4, 9].

### 2.1. Polyethylene

The use of polyethylene polymer is based on its prior extensive use and support in knee and hip arthroplasties [7, 10, 11]. Polyethylene itself is a thermoplastic polymer consisting of long hydrocarbon chains with excellent chemical resistance. Ultrahigh molecular weight polyethylene (UHMWPE) has extremely long chains with a molecular mass usually between 2 and 6 million units. The longer chains allow for more effective load transfers to the polymer backbone, resulting in a very high impact strength. UHMWPE was first used clinically in 1962 by Sir Charnley in which he incorporated a UHMWPE acetabular cup against a stainless steel femoral head for use as a total hip replacement [12].

Over the past several decades, highly cross-linked UHMWPE materials have been introduced and have become the standard of care for hip arthroplasty [7, 10]. Cross-linking of polyethylene improves wear resistance and the risk of osteolysis in the hip but comes with some concomitant decrease in mechanical properties [11].

### 2.2. Stainless Steel

Steels are alloys of iron and other elements, primarily carbon. Stainless steel is a steel alloy with a minimum of 10.5% chromium content by mass. As such, it does not readily corrode, rust, or stain as ordinary steel. Marine grade stainless 316 steel is a molybdenum-alloyed steel that is negligibly responsive to magnetic fields and is the preferred grade steel for medical implantation due to its immunity from sensitization [13].

The use of stainless steel as a material for cervical arthroplasty can be traced to the original Bristol/Cummins disc used in 1991. In the 1980s, British neurosurgeon Brian Cummins was intent on developing a solution to adjacent segment disease and collaborated with a medical engineer to create a ball-and-socket prosthetic for use in the cervical spine. This became the Bristol/Cummins disc, manufactured from 316 stainless steel at his hospital's machine shop. In all, 22 devices were implanted in a total of 20 patients and long-term follow-up out to 12 years postoperatively demonstrated these devices to still be functional [14–16].

Though stainless steel has long been used as surgical implants for many orthopedic applications, it may not always be preferred in cervical arthroplasties because of its inferior mechanical properties [4]. Newer metals such as titanium (Ti) and cobalt-chrome alloy (CoCr) have improved yield strengths and are less prone to corrosion and fatigue failure [17].

### 2.3. Titanium

Titanium is a low-density transition metal with high strength and is highly resistant to corrosion. Titanium can be alloyed with multiple other metals such as iron, aluminum, vanadium, and molybdenum to create strong, lightweight alloys for use in a variety of industries. Because of its biocompatibility, it is an ideal substance for medical implantation of prosthetics and is often alloyed with 4–6% aluminum and 4% vanadium [18].

Titanium has an inherent ability for osseointegration which stems from its lower modulus of elasticity (Young's modulus) to more closely match that of bone for greater mechanical compatibility [18]. Studies have shown the capacity for bone to bond directly with pure titanium without need for an intervening membrane or scaffold [19–21]. This observation has led to the development of porous titanium spray-coatings on the outer surfaces of cervical prosthetic implants for long-term bony fixation in the cervical spine. This effect is even more significant when hydroxyapatite is applied as well [18, 21].

Titanium alloys have not been used in arthroplasty articulating components due to their poor wear characteristics [4, 18, 22, 23]. Laboratory studies have shown that implanted titanium used as a bearing surface wears down at a higher rate than either stainless steel or cobalt-chrome because of its poor abrasion resistance qualities [17, 24, 25]. The generation of polyethylene wear debris is also the greatest with titanium, and the least with cobalt-chrome [17, 24, 26–28]. Titanium is more prone to abrasive wear due to its surface oxide layer; treating the surface of titanium with nitride or diamond-like carbon, however, improves hardness and wear characteristics while still offering the same MRI imaging compatibility [4, 29, 30]. Even with this supplementation, however, its wear properties still remain inferior to cobalt-chrome or ceramic surfaces [18].

### 2.4. Cobalt-Chrome

Cobalt-chrome (CoCr) is a metal alloy of cobalt and chromium with a very high specific strength and approximately twice the stiffness of titanium [18]. The alloy composition used to make prosthetics for surgical implantation typically contains 5–7% molybdenum and is therefore sometimes referenced as being made of cobalt-chromium-molybdenum (CoCrMo). Due to their corrosion resistance and excellent biocompatibility, CoCr alloys pose little risk of irritation, allergic reaction, or immune response [31, 32]. This is in part due to the spontaneous formation of a chromium-oxide surface film during its synthesis which renders it biocompatible with physiological environments.

Harold Bohlman is credited with designing the first corrosion-resistant cobalt-chrome alloy femoral head replacement for use as a femoral head prosthetic in 1937 [33]. Since then, CoCr alloys have shown excellent wear characteristics as successfully demonstrated through their wide utilization in joint arthroplasties. Multiple studies have demonstrated CoCr's high resistance to wear especially as compared with titanium alloy [17, 27, 28]. These alloys have seen extensive use as bearing surfaces due to these proven properties [4]. CoCr is also particularly favored as a bearing surface due to reports of reduced amount of metal and polyethylene debris when compared with titanium [17, 22, 23, 34–39].

### 2.5. MRI Characteristics

All TDR designs are safe and compatible for the magnetic field of MRI scans. Their greatest effect on MRI, however, is in the potential imaging interference that they may cause from any magnetic properties of their constituent metals [40]. The interference artifact in patients with metallic implants is due to the large differences in magnetic properties of human tissue and implanted metals [41, 42]. Depending on the ferromagnetic properties of the metal, these alloys can produce a significant amount of this distortion artifact that may confound interpretation of important anatomic structures near the TDR device such as the spinal canal, neural foramen, disc spaces, vertebral bodies, and paraspinal tissues.

The polyethylene component of a cervical TDR produces no artifact due to its nonmetallic thermoplastic polymer composition. However, the more common metal components of stainless steel, titanium, and cobalt-chrome will all produce varying degrees of magnetic susceptibility artifact. Prior craniomaxillofacial studies have found titanium to be superior to both CoCr alloy and stainless steel with regard to distortion artifact in the face, head, and neck, but it was unclear if these conclusions would be applicable to spinal anatomy [43–45]. Knott et al. investigated the differences in magnetic and radiographic imaging artifact in posterior spinal instrumentation containing stainless steel, titanium, or CoCr alloy [46]. They found that stainless steel implants produced the most artifact but that there were no significant differences in diagnostic evaluation between titanium and CoCr alloy as evaluated by a radiologist and orthopedic surgeon using a 3.0 Tesla magnetic resonance scanner.

## 3. Bearing Types

Most TDR designs utilize bearings that are configured with ball-and-socket surfaces which then articulate with each other to provide motion. The mechanical load transfer through this joint, however, leads to friction which can lead to implant fixation failure as well as the generation of wear debris [8, 47]. The choice of materials for these TDR bearing surfaces then continues to be an area of extreme importance to minimize the friction between the two bearing surfaces and decrease these risks [47, 48]. The type of bearing used in the majority of TDRs currently is either that of a metal-on-polymer design or that of a metal-on-metal design [7].

### 3.1. Metal-on-Polymer

Bearing surface technology for total joint arthroplasties traces their origins to the hip and has evolved over decades of major industrial and scientific advancement [10, 49]. Sir Charnley developed the initial hip arthroplasty which used a metal femoral head that articulated with a high-density polyethylene cup inserted into the acetabulum [47, 50]. This allowed for his idea of a “low friction arthroplasty” to counteract what he saw as unacceptable levels of frictional torque of existing metal-on-metal articular designs of that time. The standard contemporary total hip arthroplasty bearings now are based on a metal-on-polymer design utilizing a CoCr alloy femoral head which articulates with a UHMWPE acetabular socket.

Metal-on-polymer articulations have the foundation of extensive clinical experience and literature support as a bearing surface for multiple joints [10, 49]. The majority of cervical TDR implants that are FDA-approved for use in the United States incorporate iterations of metal alloy-based superior and inferior prosthetic endplates which articulate with a central polymer core.

### 3.2. Metal-on-Metal

Metal-on-metal articulation designs were initially thought to be a viable alternative to metal-on-polymer devices to reduce long-term wear. These bearing designs also generate less friction on movement which can lower the volume of wear debris as compared to polyethylene type articulations, potentially reducing local inflammation and osteolysis [6].

The metal-on-metal bearing design was first widely used in the early 1960s with total hip arthroplasty via the McKee-Farrar prosthesis. This first generation total hip replacement utilized cobalt-chrome metal bearing surfaces on both the femoral and acetabular components [51]. Although early results were favorable, this design became unpopular due to possible metal hypersensitivities and ion toxicities [49, 52–54]. Newer contemporary metal-on-metal hip articulations were subsequently developed with different CoCr alloys, but these have also come under recent scrutiny due to unexpected failures and accelerated wear [6, 38, 55–57].

Because the biomechanical forces seen in the hip joint differ than those in the spine with regard to load as well as bearing surface conformational constraints, it is still unclear how these metal-on-metal designs will truly translate with long-term use in the cervical compartment [49].

## 4. Cervical Prosthetic Devices

There are currently seven cervical artificial disc replacements that are approved for use in the United States by the Food and Drug Administration (FDA). Their materials and bearing types will be discussed further here (Table 1).

### 4.1. Medtronic Prestige ST/Prestige LP

The original Medtronic Prestige ST (Medtronic Sofamor Danek, Memphis, TN, USA) artificial disc utilizes a superior stainless steel convex ball that articulates with an inferior stainless steel concave trough. This design built upon and refined the original Bristol/Cummins artificial disc, replacing the inferior hemispherical cup with a shallow ellipsoidal saucer to permit more translation. Since that metal-on-metal original design, the product has undergone multiple evolutions with the FDA recently approving the Prestige LP device in 2014 for use here in the United States. The Prestige LP retains the same ball-and-trough socket design but the implant itself utilizes a proprietary titanium-ceramic composite material. A plasma-spray titanium coating on the outer surface encourages bony growth into the device.

### 4.2. Depuy-Synthes ProDisc-C

The Depuy-Synthes ProDisc-C (Synthes Spine, Paoli, PA, USA) prosthesis is an adaptation using the same design as the lumbar total disc replacement ProDisc-L created by the same company. The articular surfaces used include a UHMWPE inlay ball locked into the inferior endplate which articulates with a CoCr alloy socket in the superior endplate. The outer coating incorporates a porous plasma-sprayed titanium coating to encourage bony growth for long-term stability.

### 4.3. Medtronic Bryan

The Medtronic Bryan (Medtronic Sofamor Danek, Memphis, TN, USA) artificial disc consists of a pair of superior and inferior identical titanium (Ti) shells that conform to and articulate with a central polycarbonate urethane (PCU) core. A flexible polyether urethane (PEU) sheath then surrounds the core to prevent tissue ingrowth into the articulating surfaces.

Titanium alloy seal plugs help to retain a sterile saline lubricant. Long-term stability of the implant within the cervical spine is achieved with bony growth into porous-coated titanium alloy end plates.

### 4.4. LDR Mobi-C

The LDR Mobi-C cervical artificial disc (LDR USA, Austin, TX, USA) uses a three-piece design with CoCr alloy superior and inferior endplates which encase a UHMWPE mobile bearing core insert. The endplates themselves are coated with a plasma-sprayed titanium and hydroxyapatite coating for long-term fixation within the cervical spine.

### 4.5. Globus SECURE-C

The Globus SECURE-C prosthesis (Globus Medical, Audubon, PA, USA) also uses a three-piece design with two CoCr alloy endplates. The UHMWPE central core articulates with the superior CoCr endplate via a spherical surface and interfaces with the inferior CoCr endplate through a cylindrical surface. The outer portions of the CoCr alloy endplates use a titanium plasma-spray coating for bony ongrowth.

### 4.6. NuVasive PCM

The PCM (porous-coated motion) cervical artificial disc was initially developed by Cervitech (Cervitech, Rockaway, NJ, USA) and subsequently acquired by NuVasive (NuVasive, San Diego, CA, USA). The PCM disc replacement's superior and inferior endplates are made entirely of CoCr alloy while a UHMWPE central core is locked into the inferior endplate for articulation with the superior endplate. The articulating surface extends across the entire bearing and allows for a larger radius of movement and increased translation during the rotational arc. A titanium calcium phosphate coating is electrochemically applied to the outer surface of the superior and inferior CoCr alloy endplates to allow for bony growth by the vertebral bodies into the prosthesis.

## 5. Adverse Biologic Effects

Although all cervical prosthetics are constructed with materials that are biocompatible for implantation, the long-term effects of these materials have become more clinically relevant as more follow-up has been achieved. Wear debris is generated over time as the articulating surfaces move against each other, and this debris can subsequently lead to multiple adverse effects including inflammatory hypersensitivity reactions, pseudotumor formation, osteolysis, and implant loosening.

### 5.1. Wear Debris

Generation of wear debris in artificial joints has been shown to be the primary source of implant degradation, and the subsequent tissue and inflammatory reaction to the debris significantly limits the longevity of the prosthesis [4]. This debris has been associated with osteolysis, implant loosening, and subsequent prosthesis failure [4, 37, 38]. Polyethylene-on-metal provides a low friction surface contact but generates polyethylene wear debris that in the literature has been established as a cause of hip and knee arthroplasty failure. Cross-linking with gamma irradiation has been used to improve those properties in ultrahigh molecular weight polyethylene but with some effect to its mechanical properties [58, 59]. Metal-on-metal articulations lower wear rates dramatically but still generate a lower volume through higher quantity of smaller particle debris. Metal-on-metal designs also provide less shock absorption than metal-on-polyethylene [4].

Host reaction to wear debris is related to particle shape, quantity, volume, and concentration [49, 60]. Metal-on-metal articulations produce a predominance of needle-shaped particles which have been associated with greater inflammation from prior observations in polyethylene debris [61–64]. Wear debris from metal-on-metal bearings can also form corrosion products and molecular complexes [49, 65, 66]. Wear debris particles are readily phagocytosed by inflammatory cells, which in turn trigger proinflammatory cascades and oxidative stress. An individual patient's response to this debris is also unpredictable, with some tolerating it well and others poorly [67, 68]. All bearing surfaces will produce wear debris, but poorly positioned or otherwise compromised prosthetics may produce further pathologic wear [49, 69, 70].

### 5.2. Immune Response

Polyethylene materials have been used for decades and have since become the standard of care in hip arthroplasty [10]. However, it has long been known that wear debris can have an effect on the local periprosthetic tissue. In one of the only long-term studies of retrieved explanted TDR tissue, Kurtz et al. noted a chronic inflammatory response in the periprosthetic fibrous tissues from 15 of 16 patients who had undergone revision lumbar surgery for removal of the TDR prosthetic. Examination of this tissue demonstrated lymphocytes, macrophages, and giant cells, which all had ingested small polyethylene particles. Greater implantation time was associated with greater presence of wear debris and giant cells, accompanied by inflammatory cytokines. Innervation and vascularization were also noted in the tissue, suggestive of the development of neuroinflammatory-induced pain in these TDR patients [7, 48]. They concluded that wear debris from TDR initiated a complex interaction within the periprosthetic tissue of the spine and pointed to the subsequent inflammatory cascade as a potential etiology of postoperative intractable pain even out to 16 years from implantation.

Veruva et al. recently reviewed the literature with regard to biomaterials that can affect wear on performance on TDR [6]. They focused on implant wear and any periprosthetic tissue inflammation as a response to implantation of the prosthetic device. In their review of papers describing devices and tissues after explanation, they found that wear-associated complications may be specific to the biomaterial selection for TDR. For metal-on-polymer prosthetics in the cervical spine, small and large polymeric debris was generated which triggered an innate immune response with nearby tissue activation of macrophages and giant cells. For total hip arthroplasties (THA), polyethylene wear and its subsequent innate inflammatory response have been associated with osteolysis, aseptic loosening, and clinical failure [6, 10, 38]. In the spine, vertebral osteolysis seems to be a rare event [71]. This is even after Punt et al. observed on the order of 1 billion polyethylene particles per gram of explanted periprosthetic tissues from TDR patients [7, 72]. Metal-on-metal prosthetics created small metallic wear debris which triggered an adaptive immune response of activated lymphocytes. This wear process poses the risk of metallosis, pseudotumors, aseptic vasculitis, and metal hypersensitivity [6, 49, 73, 74]. Fretting and corrosion products were seen in some metal-on-metal cervical TDR but their clinical effect was unclear [7, 75].

A tremendous soft tissue reaction has been observed to occur in rare occasions with metal-on-metal implants. Termed a pseudotumor, this adverse reaction can cause significant mass effect on neighboring structures and in the hip arthroplasty literature has been shown to cause pain, nerve palsy, joint dislocation, metal hypersensitivity reactions, and osteolysis [49, 76–78]. One case report on a cervical device implanted investigationally described the formation of pseudotumor at the C4-5 disc space extending ventrally down to the midbody of C6 [73]. On explanation of the implant, a large yellowish necrotic mass was discovered extending down into the ventral spinal canal. Histology demonstrated a large area of necrotic debris with prominent lymphocytic infiltrate. The Medtronic Prestige ST artificial disc is currently the only FDA-approved cervical TDR device that utilizes a metal-on-metal design using stainless steel.

Metal hypersensitivity reactions after metal-on-metal bearing device implantations are presumed to be due to type IV-delayed hypersensitivities based on their immunohistochemical features [56]. The inflammatory response seen is chronic and composed of mononuclear phagocytes, without an acute inflammatory character due to very few neutrophils observed in the tissue. Some reports exist to suggest that hypersensitivity to the metal-on-metal wear debris is the underlying pathophysiology of failed implantation, though evidence is conflicting [57, 79, 80]. Metal degradation wear debris has not been noted so far to have been associated with necrosis or tissue degeneration in the spine [7, 72].

### 5.3. Osteolysis

Long-term complications such as wear debris-induced osteolysis are well documented in the large joint arthroplasty literature [81–83]. Periprosthetic bone loss following hip arthroplasty placement accounted for over 75% of patients undergoing revision hip surgery in one study and in many other studies accounts for greater than the sum of all other complications [84, 85]. In most series beyond 10 years, the reported prevalence of aseptic osteolysis of hip implants is between 32 and 62% [51, 86–89].

There is also evidence to support this complication in the cervical spine. The available literature at this time of osteolysis in cervical TDR has not been robust enough to draw conclusions for a predicted incidence of this long-term condition beyond just several reported cases, both infectious and aseptic [7, 83]. Hacker et al. reported 4 patients with either the Bryan or Prestige LP discs who experienced periprosthetic bone loss after a minimum of 4 years of follow-up [83]. In one patient who was presumed to have an infection, there was marked loss of vertebral body bone with deformity. Review of the explanted device and tissue was suggestive of a low virulence bacterial infection based on the appearance of macrophages, but no agent was identified and all cultures had resulted negative. Although they did not identify any convincing evidence for osteolysis as a cause for the bone loss in these patients, they acknowledged that its potential must exist based on prior experience with arthroplasty devices. Tumialán and Gluf also reported a case of osteolysis with the ProDisc-C device in a patient who developed progressive neck pain at 9 months of follow-up [71]. Imaging at 9 and 15 months demonstrated a progressive osteolytic process which prompted explanation and conversion to an arthrodesis. Further follow-up after the arthrodesis showed resolution of the osteolytic process and radiographic fusion.

The most widely accepted mechanism of osteolysis involves implant particulate wear debris of any material which then promotes inflammation that causes long-term tissue damage and bone erosion leading to implant loosening [87, 90]. At the cellular level, proosteoclastic inflammatory mediators stimulate differentiation of osteoclasts which subsequently mediate bone resorption leading to failure of the prosthesis [67].

### 5.4. Adverse Events

The current literature is fairly sparse with regard to dedicated reports of adverse events after cervical TDR that could be directly attributable to material wear, immune response hypersensitivity, or osteolysis. Hacker et al. reported on two patients who presented with neck pain due to periprosthetic bone loss around their Bryan discs but could not conclude definitively that the bone loss was from osteolysis instead of another etiology [83]. Neither patient required further intervention and one patient clinically improved at 1-year follow-up. Guyer et al. reported on one case of a patient, implanted with a Kineflex-C (Spinal Motion Inc, Mountainview, CA) cervical arthroplasty device, who was subsequently found on CT-myelography to have a soft-tissue mass causing canal stenosis [74]. The implant required a reoperation for implant removal, arthrodesis, and soft tissue mass resection. Analysis of the soft tissue mass suggested a delayed-type hypersensitivity to metal that resulted in the chronic inflammatory pseudotumor. Tumialán and Gluf reported on one patient who was found to have progressive vertebral body osteolysis after implantation of a ProDisc-C that resulted in persistent radicular and axial neck pain and required reoperation with removal of the implant and arthrodesis at that level [71]. The patient's pain improved postoperatively and the authors concluded that the osteolysis was likely due to an immune-mediated metal hypersensitivity response which resolved upon removal of the implant. Zigler et al., however, reported on the 5-year results of 103 patients treated with the ProDisc-C and found no adverse events related to polyethylene wear, osteolysis, or material failure [91]. Likewise, Sasso et al. reported on the 4-year results of 242 patients treated with the Bryan disc and similarly found that no arthroplasty device required removal for wear or wear-related failure [92]. The existing literature would suggest that adverse events related to material wear are suitably rare, although further research and follow-up are needed to better delineate the risks of these occurrences.

### 5.5. Clinical Evaluation

Maintaining a high level of clinical suspicion for the adverse biological effects of material wear is important for diagnosis and treatment. The proinflammatory cascade that results from significant wear debris may result in a type of neuroinflammatory-induced pain at the site of the prosthesis that may be severe enough to necessitate removal of the implant. This inflammatory process may also cause pseudotumor formation that, if large enough, can lead to the clinical spectrum and manifestations of cervical radiculopathy or myelopathy. Both pseudotumor and osteolysis can cause implant loosening and hardware failure, which will possibly lead to segmental instability and axial mechanical pain. Persistent postoperative neck or arm pain should prompt further evaluation with dynamic radiographs, CT, or MRI to allow for further workup of these material-related complications. Knowledge of these potential clinical findings in routine follow-up will assist the surgeon in capturing these complications early for further management and surgical treatment as needed.

## 6. Conclusion

Cervical TDR is a motion-sparing operation that provides a surgical alternative to fusion for selecting patients with cervical radiculopathy or myelopathy. Knowledge and understanding of the variety of biomaterials available will ensure the continued development of safe and effective prosthetics with increased longevity and decreased biological effects over a lifetime. An appreciation of material wear characteristics will help the surgeon maintain a high clinical suspicion of postoperative clinical manifestations of material-related biological effects.

## Figures and Tables

**Table 1:** Cervical artificial disc replacements FDA-approved for use in the United States.

Device	Manufacturer	Bearing type	Materials	Year FDA-approved
Prestige ST	Medtronic	MoM	Stainless steel	2007
ProDisc-C	Depuy-Synthes	MoP	CoCr, UHMWPE	2007
Bryan	Medtronic	MoP	Ti, PCU	2009
SECURE-C	Globus	MoP	CoCr, UHMWPE	2012
PCM	NuVasive	MoP	CoCr, UHMWPE	2012
Mobi-C	LDR	MoP	CoCr, UHMWPE	2013
Prestige LP	Medtronic	MoM	Ti-ceramic	2014

FDA: Food and Drug Administration; MoM: metal-on-metal; MoP: metal-on-polymer; CoCr: cobalt-chromium alloy; UHMWPE: ultrahigh molecular weight polyethylene; Ti: titanium alloy; PCU: polycarbonate urethane.
